# An improved DNA-binding hot spot residues prediction method by exploring interfacial neighbor properties

**DOI:** 10.1186/s12859-020-03871-1

**Published:** 2021-05-17

**Authors:** Sijia Zhang, Lihua Wang, Le Zhao, Menglu Li, Mengya Liu, Ke Li, Yannan Bin, Junfeng Xia

**Affiliations:** 1 Key Laboratory of Intelligent Computing and Signal Processing of Ministry of Education, Institutes of Physical Science and Information Technology, Anhui University, Hefei, 230601 Anhui China; 2 Key Laboratory of Computational Neuroscience and Brain-Inspired Intelligence (Fudan University), Ministry of Education, Shanghai, China

**Keywords:** Protein–DNA complex, Hot spot, Interfacial neighbor property, Support vector machine, Feature selection

## Abstract

**Background:**

DNA-binding hot spots are dominant and fundamental residues that contribute most of the binding free energy yet accounting for a small portion of protein–DNA interfaces. As experimental methods for identifying hot spots are time-consuming and costly, high-efficiency computational approaches are emerging as alternative pathways to experimental methods.

**Results:**

Herein, we present a new computational method, termed inpPDH, for hot spot prediction. To improve the prediction performance, we extract hybrid features which incorporate traditional features and new interfacial neighbor properties. To remove redundant and irrelevant features, feature selection is employed using a two-step feature selection strategy. Finally, a subset of 7 optimal features are chosen to construct the predictor using support vector machine. The results on the benchmark dataset show that this proposed method yields significantly better prediction accuracy than those previously published methods in the literature. Moreover, a user-friendly web server for inpPDH is well established and is freely available at http://bioinfo.ahu.edu.cn/inpPDH.

**Conclusions:**

We have developed an accurate improved prediction model, inpPDH, for hot spot residues in protein–DNA binding interfaces by given the structure of a protein–DNA complex. Moreover, we identify a comprehensive and useful feature subset including the proposed interfacial neighbor features that has an important strength for identifying hot spot residues. Our results indicate that these features are more effective than the conventional features considered previously, and that the combination of interfacial neighbor features and traditional features may support the creation of a discriminative feature set for efficient prediction of hot spot residues in protein–DNA complexes.

**Supplementary information:**

**Supplementary information** accompanies this paper at 10.1186/s12859-020-03871-1.

## Background

Protein–DNA interactions are fundamental to almost all biological processes, such as DNA replication and gene regulation [1]. Previous studies have revealed that the distribution of binding energy of proteins is not average among the interaction surfaces [2, 3]. Only a small and complementary set of interface residues termed hot spots contribute mainly to the binding free energy. It is crucial to identify hot spots for understanding the underlying biological mechanism of protein–DNA interaction [4] and their role in cancer [5, 6]. Experimental methods like alanine scanning mutagenesis have been applied to investigate the DNA-binding hot spots [7]. As experimental technique for identifying hot spots is inefficient and labor-intensive, there is a need for developing computational approaches to predict hot spots.

Several computational methods have been developed to identify hot spots in protein–DNA complexes. One class is based on molecular mechanics such as called SAMPDI [8] and PremPDI [9], which predict protein–DNA binding free energy changes upon missense residue mutations. And a graph-based method termed mCSM-NA [10] can predict the effects of single amino acid mutations on protein-nucleic acid affinity. These methods have achieved comparable results in predicting hot spot residues in protein–DNA interfaces. However, these predictors require a high quality of input structures because their predictions are based on the simulation of protein structures. In our pervious feature-based approach PrPDH [11], we used support vector machine (SVM) and 10 selected optimal features to boost the prediction performance of DNA-binding hot spots.

In this study, we developed an improved structure-based protein–DNA hot spot prediction model termed inpPDH, which integrated traditional properties used in previous hot spot prediction tasks [12–15] and the new interfacial neighbor properties (INPs). From these features, a comprehensive and powerful feature subset was selected using a two-step feature selection method. Based on the selected features, a SVM classifier was built for prediction. Empirical studies show that our method achieves generally better performance in predicting hot spots compared to the state-of-the-art predictors. A web server of inpPDH is available at http://bioinfo.ahu.edu.cn/inpPDH.

## Results and discussion

### Evaluation of two-step feature selection method

The feature selection we used in this study is a two-step strategy. We applied SVM-RFE as the first step feature selection and obtain 11 features. As reported that SVM-RFE usually provides a criterion to rank features based on their relevancy and complementarity but does not take the redundancy among features into account [16], we therefore implemented the second step to remove potential redundant features of high correlations. We calculated the Pearson correlation coefficients among 11 features and removed potential redundant features with a threshold of 0.65. Finally, an optimal group of 7 features were produced by performing this two-step feature selection method.

**Fig. 1:**
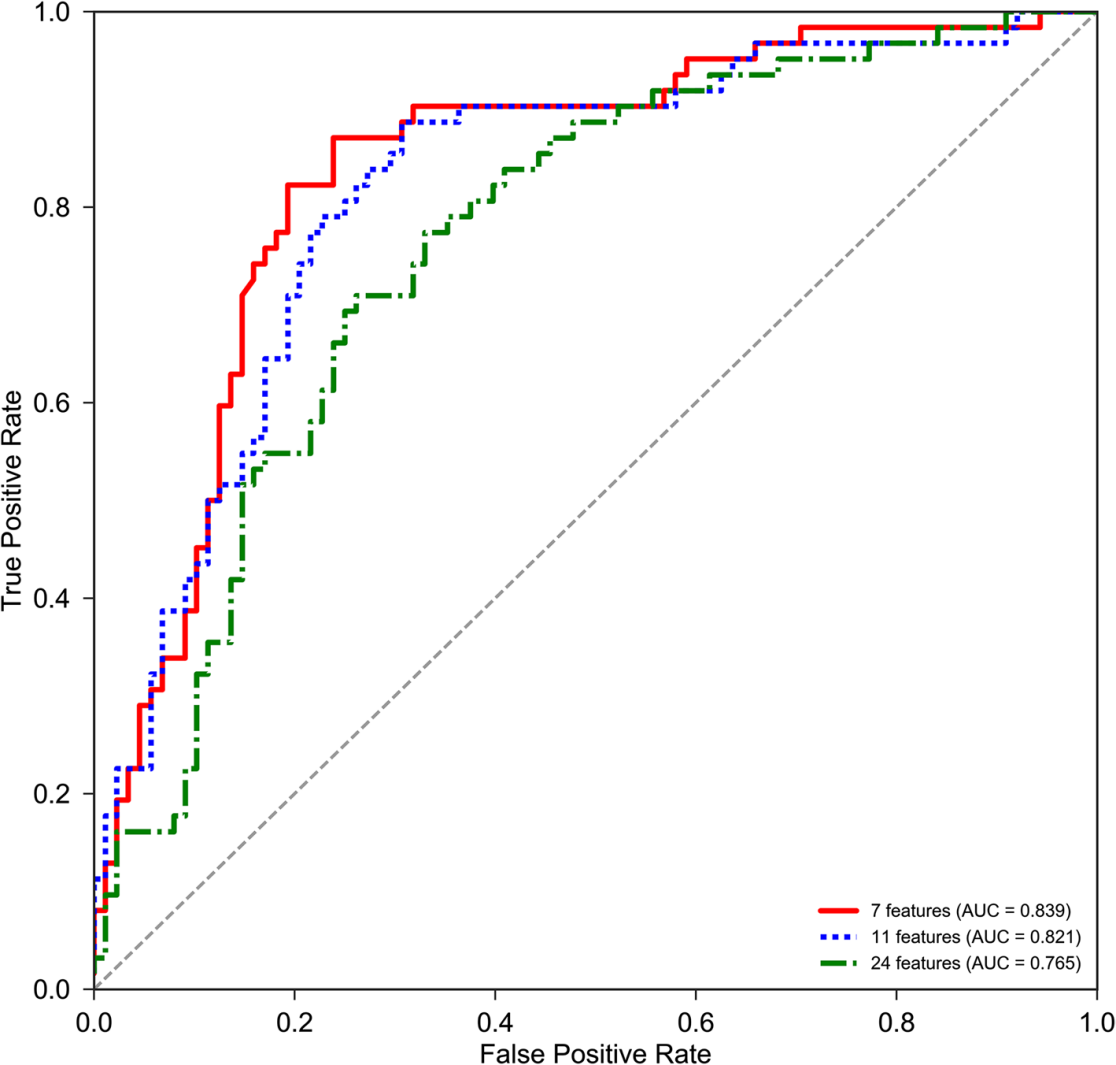
The ROC curves of the model with raw 24 features, one-step 11 features and two-step 7 features on the training set

Figure 1 shows the performance comparison before and after feature selection, where 24 features represent the model without feature selection, 11 features represent the model with one-step feature selection and 7 features indicates the model with two-step feature selection. As we can see, the model reaches the highest AUC score with 0.839 after performing two-step feature selection. Compared with one-step 11 features and raw 24 features, the AUC score has been increased $\mathrm{with}$ 0.018 and 0.074, respectively.

**Fig. 2:**
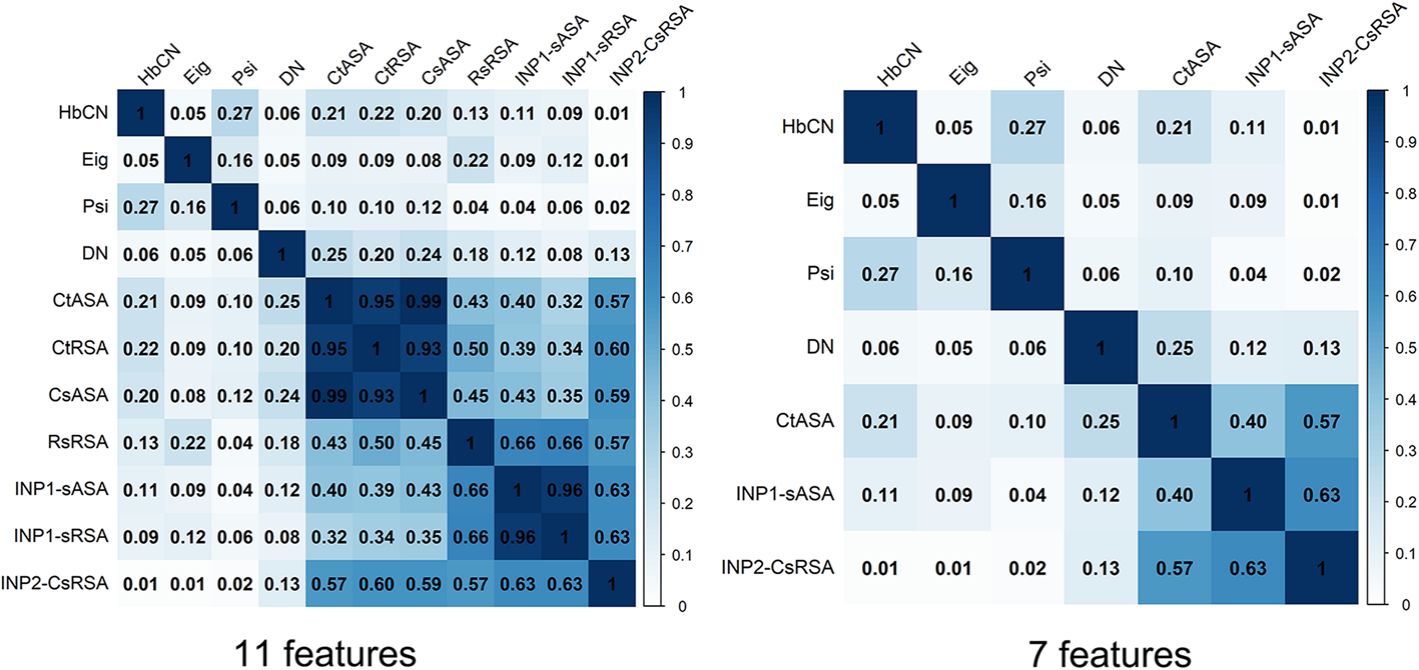
The correlation heat map of one-step 11 features and two-step 7 features. The lowest correlation coefficient to the highest correlation coefficient is represented by the number 0–1. The number in each block is the correlation coefficient of two features

We further evaluated the correlation coefficients among one-step 11 features and two-step 7 features. The correlation heat map of these two feature subsets is shown in Fig. 2. It is obvious that 4 pairs of features where the correlation coefficients are more than 0.9 among 11 features. And for the two-step selected 7 features, all of correlation coefficients are under 0.65. It also shows that the correlation coefficients between the features based on ASA and INP are generally higher than correlation coefficients between the other features. In addition, these features such as Psi (IUPAC peptide backbone torsion angles PSI) and Eig (Eigenvector centrality index) are lowly correlated with the features based on ASA and INP. Therefore, we inferred that there exists complementarity among these features. In summary, we concluded that the two-step feature selection can achieve a greater performance with minimum redundancy.

### Assessment of feature importance

In this study we proposed two kinds of interfacial neighborhood properties (INPs) based on ASA and CASA, and obtained a total of 8 INPs. Among the selected 7 optimal features, 2 of them (INP1-sASA and INP2-CsRSA) are newly encoded. To better understand the relative contributions of these features used within inpPDH and to explore the relative importance of each feature, we compared inpPDH’s cross-validation performance leaving out each feature from the analysis (Table 1). Removing INP1-sASA causes inpPDH’s performance to drop significantly, which emphasizes the importance of this feature. And the following ones are Psi and INP2-CsRSA. In addition, these two features show more contributions in correctly predicted hot spot residues, with ΔSEN of 0.178 and 0.081, respectively. The feature of half-sphere C_α_–C_β_ contact numbers (HbCN) does not substantially affect performance. We conclude that the two newly encoded INP features make an obvious improvement on prediction model by their individual and cooperative roles in two-step selected 7 features.

**Table 1:** The evaluation of single feature performance on the training set

Features	SEN	SPE	PRE	F1	MCC	ACC	AUC
All features	0.726	0.841	0.763	0.744	0.571	0.793	0.839
no INP1-sASA	0.661	*0.761*	0.661	0.661	0.423	0.720	*0.777*
no Psi	*0.548*	0.841	0.708	*0.618*	0.411	0.720	0.780
no INP2-CsRSA	0.645	*0.761*	*0.656*	0.650	*0.408*	*0.713*	0.796
no Eig	0.645	0.784	0.678	0.661	0.433	0.727	0.809
no CtASA	0.661	0.795	0.695	0.678	0.460	0.740	0.807
no HbCN	0.742	0.795	0.719	0.730	0.535	0.773	0.826
no DN	0.694	0.830	0.741	0.717	0.529	0.773	0.811

The lowest value in each column is shown in italics

### Comparison with other methods

To further verify the performance of our model, we compared its performance with the state-of-art methods, including binding affinity change predictors (SAMPDI, PremPDI and mCSM-NA) and our previous method (PrPDH). We obtained the prediction results by submitting the test set to the web servers of these methods. The results are displayed in Table 2. Our method inpPDH shows high success rates in contrast to the other four methods. The AUC value of our method is 0.833, while the other methods have AUC values in the range of 0.661–0.764. Therefore, our method can effectively distinguish hot spots from non-hot spots in protein–DNA interfaces. Our method can correctly predict hot spots from the data set with SEN = 0.731 and PRE = 0.731, which means that inpPDH can correctly predict 73.1% of the true hot spots from this data set (sensitivity), and 73.1% of the predicted hot spots are identified as true hot spots (precision). Our previous method PrPDH efficiently identified non-hot spots (SPE = 0.816), while it could not correctly identify hot spots (SEN = 0.692) compared with inpPDH. The AUC value of inpPDH is 6.9 percentage points higher than that of PrPDH (the detailed prediction results of each method can be found in Additional file 1). In addition, inpPDH’s other measures, F1 score (0.731), MCC (0.547), and ACC (0.781) are still competitive among all tested methods. We further performed statistical analysis to show whether the difference from these comparisons is statistically significant or not. Specifically, we randomly selected the test set ten times to get ten balanced subsets, with 20 hot spots and 20 non-hot spots respectively. We calculated the AUC values for these methods in each subset, and the p-value of AUC between inpPDH and other methods [17]. It can be observed that inpPDH has outperformed other methods with the p-values much smaller than 0.05. From these analyses, we can see that our feature-based method gives remarkably better prediction performance in comparison to other available approaches for predicting DNA-binding hot spot residues.

**Table 2:** Performance comparisons of our method with other methods on the test set

Tools	SEN	SPE	PRE	F1	MCC	ACC	AUC	*p* value
inpPDH	*0.731*	*0.816*	*0.731*	*0.731*	*0.547*	*0.781*	*0.833*	*#*
PrPDH	0.692	0.816	0.720	0.706	0.511	0.766	0.764	4.130e−05
SAMPDI	0.654	0.658	0.567	0.607	0.307	0.656	0.690	8.772e−12
PremPDI	0.577	0.737	0.600	0.588	0.316	0.672	0.708	8.015e−08
mCSM-NA	0.538	0.737	0.583	0.560	0.279	0.656	0.661	1.988e−17

The highest value in each column is shown in italics. #indicates the reference item for calculating the *p* value with other methods

## Conclusions

As only several studies have been published to investigate DNA-binding protein hot spot, there is a need for developing more accurate and efficient computational method to predict hot spot residues. In this study, we proposed a feature-based method called inpPDH to distinguish hot spots from protein–DNA interface residues. The performance of our model inpPDH was first evaluated by the tenfold cross validation and further validated with an independent test set. Clearly, our method can provide favorable performance compared with the existing hot spot prediction methods. Moreover, we developed two kinds of interfacial neighbor properties based on ASA features and the results show that these interfacial neighbor properties are effective in describing the differences and contributing to the protein–DNA binding events. We believe that inpPDH can be a useful tool for accurately identifying DNA-binding hot spots and a web server implementation is freely available at http://bioinfo.ahu.edu.cn/inpPDH.

In our future work, on one hand we will try to develop more sophisticated prediction methods based on advanced machine learning methods such as deep learning methods, and on the other hand, we will explore more characteristic features that better describe the different energetic contributions of the protein–DNA interface residues.

## Materials and methods

### Data sets

The data sets used in this study are the same as that used by our previous work, PrPDH [11]. We collected 108 protein–DNA complexes from dbAMEPNI [18] and SAMPDI [8] and removed the redundant sequences to ensure the similarity of any two protein sequences no more than 40%. By these processes, we obtained a data set of 64 complexes including 88 hot spots and 126 non-hot spots. These complexes were randomly divided into a training set (40 complexes) and a test set (24 complexes). The final training set consists of 62 hot spots and 88 non-hot spots and the final test set includes 26 hot spots and 38 non-hot spots.

### Feature representation

To build a predictor that can distinguish hot spots from non-hot spots, we generated a total of 24 features including sequence-based and structure-based features to test feature selection method and train our model. A detailed list of these 24 candidate features can be found in Table 3. Note that the first 4 features and the 13th, 14th and 15th features in the table have showed effective performance for correctly predicting hot spot residue, which have used as part of feature set in our previous work [11]. The remaining 17 features are new features proposed in this study. More detailed descriptions of these features are shown below.

**Table 3:** Summary of the features used in this study

Number	Symbol	Feature description
1	BsASA	Bound side-chain absolute ASA
2	BsRSA	Bound side-chain relative ASA
3	BtASA	Bound total absolute ASA
4	BtRSA	Bound total relative ASA
5	CsASA	Change in side-chain absolute ASA upon complexation
6	CsRSA	Change in side-chain relative ASA upon complexation
7	CtASA	Change in total absolute ASA upon complexation
8	CtRSA	Change in total relative ASA upon complexation
9	RsASA	Relative change in side-chain absolute ASA upon complexation
10	RsRSA	Relative change in side-chain relative ASA upon complexation
11	RtASA	Relative change in total absolute ASA upon complexation
12	RtRSA	Relative change in total relative ASA upon complexation
13	Eig	Eigenvector centrality index
14	Psi	IUPAC peptide backbone torsion angles PSI
15	HbCN	Half-sphere Cα-Cβ contact numbers
16	DN	The number of hydrogen bond donor residues
17	INP1-sASA	Interfacial neighborhood property 1 based on side-chain absolute ASA
18	INP1-sRSA	Interfacial neighborhood property 1 based on side-chain relative ASA
19	INP1-tASA	Interfacial neighborhood property 1 based on total absolute ASA
20	INP1-tRSA	Interfacial neighborhood property 1 based on total relative ASA
21	INP2-CsASA	Interfacial neighborhood property 2 based on change in side-chain absolute ASA upon complexation
22	INP2-CsRSA	Interfacial neighborhood property 2 based on change in side-chain relative ASA upon complexation
23	INP2-CtASA	Interfacial neighborhood property 2 based on change in total absolute ASA upon complexation
24	INP2-CtRSA	Interfacial neighborhood property 2 based on change in total relative ASA upon complexation

#### Solvent accessible surface area

From previous studies, we have learned that solvent accessible surface area (ASA) features are discriminative and effective to distinguish DNA-binding residues from non-binding residues on surface of DNA-binding residues [19]. We employed the program NACCESS [20] to calculate the absolute ASA and relative ASA (RSA) for every interface residue. From ASA and RSA, we extracted two attributes: total (the sum of all atom values) and side-chain (the sum of all side-chain atom values). The CASA, or the ASA change of a residue upon protein–DNA complex formation (bound) from monomer state (unbound), are calculated as follows: CASA(*i*) = ASAmono(*i*) − ASAcomp(*i*), where ASAmono(*i*) and ASAcomp(*i*) are the ASA of the target interface residue *i* in monomer and complex, respectively. We also calculated the CRSA (the RSA change of a residue upon complexation) with the same equation. Moreover, the relative changes of absolute ASA (RASA) and relative ASA (RRSA) between the unbound and bound states of the residues were calculated as in our previous work [13]: RASA(*i*) = CASA(*i*)/ASAmono(*i*), RRSA(*i*) = CRSA(*i*)/RSAmono(*i*). Therefore, there are 12 different ASA features (Table 3).

#### Eigenvector centrality index

The analyses of amino acid network could help reveal the functional region, structure, stability and folding of proteins [21] and the nodes in amino acid network represent the interface residues and the edges are the interactions between each two residues. To measure the influence of a node in the network, we calculated the eigenvector centrality index using the Network Analysis of Protein Structures (NAPS) [22] program.

#### Backbone angles and contact numbers

In this study, we used Definition of Secondary Structure of Proteins (DSSP) [23] to calculate the peptide backbone torsion angle PSI, and we computed the contact numbers of C_α_-C_β_ in half sphere using SPIDER3 [24].

#### Hydrogen bond

We calculated the number of hydrogen bonds of donor residues in bound status using HBPLUS [25].

#### Interfacial neighborhood properties

Existing methods generally predict whether a given residue is likely to be a hot spot by extracting features only from the target residue itself, which cannot represent the real situation well. With this in mind, we defined two kinds of interfacial neighborhood properties (INPs) based on the ASA and CASA features for each target residue *i*, and 8 INP features (Table 3) were generated by the equations below:

1 $$\mathrm{INP}1\left(i\right)=\frac{{\mathrm{ASA}}_{\mathrm{mono}}\left(i\right)}{\frac{1}{n}\sum_{j=1}^{n}{\mathrm{ASA}}_{\mathrm{mono}}\left(j\right)}-\frac{{\mathrm{ASA}}_{\mathrm{comp}}\left(i\right)}{\frac{1}{n}\sum_{j=1}^{n}{\mathrm{ASA}}_{\mathrm{comp}}\left(j\right)}$$

2 $$\mathrm{INP}2\left(i\right)=\frac{\mathrm{CASA}(i)}{\frac{1}{n}\sum_{j=1}^{n}\mathrm{CASA}(j)}$$

where *j* is the target residue’s neighbor residue located within a sphere of 6.5 Å [12] of C_α_ atoms on the interface, and *n* is the total number of neighbor residues.

### Two-step feature selection

For data set with small size used in this study, excessive features are more likely to cause overfitting. Here, we implemented a two-step feature selection strategy to remove potentially redundant features. In the first step, we employed SVM-based recursive feature elimination (SVM-RFE) [26] to filter features with bad performance. SVM-RFE is a wrapper-based method which uses weight magnitude as the ranking criterion to evaluate the importance of each feature. For every iteration, it excludes the last-ranked feature and the training process stops until yielding the best performance. In the second step, we calculated the Pearson correlation coefficient among the selected features from the first step and removed potential redundant features with a highly positive correlation threshold 0.65 based on our previous study [27].

### Model construction

As a widely used machine learning algorithm, SVM has an ability to achieve favorable classification results on the training set with small size [28]. We have compared the SVM in our previous work [11] with other classification algorithms such as random forest, naïve Bayes and k-nearest neighbors, and found that SVM outperformed these algorithms on both the training and test sets. So we continue to apply SVM in this work. Specifically, we applied the LIBSVM [29] with the radial basis function (RBF) kernel to construct the model. Meanwhile, tenfold cross-validation was used to design our method and approximate the prediction performance on the training data set. To improve the performance of the predictor, the capacity parameter *C* and the kernel parameter *γ* of the SVM were tried using a grid search method. We set the range of *C* from 0.1 to 10 and *γ* from 0.005 to 0.5 and used tenfold cross-validation on the training set to measure different parameters based on our previous study [30]. The optimal parameters of *C* and γ are 4.5 and 0.05, respectively.

### Evaluation criteria

To quantify the performance of our prediction method, we adopted sensitivity (SEN), specificity (SPE), precision (PRE), F1 score (F1), accuracy (ACC), and Matthews correlation coefficient (MCC) measures [31, 32] by the equations below:

3 $$SEN = TP/(TP+FN)$$

4 $$SPE = TN/(TN+FP)$$

5 $$PRE = TP/(TP+FP)$$

6 $$F1 = \frac{2\times SEN\times PRE}{SEN+PRE}$$

7 $$\mathrm{ACC}=\frac{TP+TN}{TP+TN+FP+FN}$$

8 $$MCC = \frac{TP\times TN-FP\times FN}{\sqrt{(TP+FP)(TP+FN)(TN+FP)(TN+FN)}}$$

where TP, FP, TN and FN represent the number of true positive (correctly predicted hot spot residue), false positive (non-hot spot residue incorrectly predicted as hot spot), true negative (correctly predicted non-hot spot residue) and false negative (hot spot residue incorrectly predicted as non-hot spot), respectively.

For the sake of completeness, we also plotted the receiver operating characteristics (ROC) curve to evaluate performance in this work. The normalized area under the ROC curve (AUC) can measure the classifier’s performance.

## Supplementary information

**Additional file 1: Table S1**. The detailed prediction results of inpPDH, PrPDH, SAMPDI, PremPDI and mCSM-NA on the test set

## Data Availability

The data and the tool are freely available on the website: http://bioinfo.ahu.edu.cn/inpPDH.

## Abbreviations

SVM: Support vector machine
INPs: Interfacial neighbor properties
IUPAC: Peptide backbone torsion angles
PSI: Psi
Eig: Eigenvector centrality index
HbCN: Half-sphere Cα-Cβ contact numbers
SEN: Sensitivity
SPE: Specificity
PRE: Precision
F1: F1 score
ACC: Accuracy
MCC: Matthews correlation coefficient
ROC: Receiver operating characteristics
AUC: Area under the ROC curve
ASA: Accessible surface area
RSA: Relative ASA
NAPS: Network analysis of protein structures
DSSP: Definition of secondary structure of proteins
SVM-RFE: SVM-based recursive feature elimination
RBF: Radial basis function
