# Supplementary material for: An improved DNA-binding hot spot residues prediction method by exploring interfacial neighbor properties
Source: BMC Bioinformatics. 2021 May 17;22(Suppl 3):253. doi: 10.1186/s12859-020-03871-1 (PMC8130120; doi:10.1186/s12859-020-03871-1)
Supplement: Supplementary file 1 — Additional file 1: Table S1. The detailed prediction results of inpPDH, PrPDH, SAMPDI, PremPDI and mCSM-NA on the test set [file 12859_2020_3871_MOESM1_ESM.docx]

**Table S1. The detailed prediction results of inpPDH, PrPDH, SAMPDI, PremPDI and mCSM-NA on the test set.**

|  | inpPDH | PrPDH | SAMPDI | PremPDI | mCSM-NA |
| --- | --- | --- | --- | --- | --- |
| 1AAY_R118A | 0.784 | 0.753 | 1.878 | 2.780 | -3.944 |
| 1AAY_D120A | 0.186 | 0.544 | 1.564 | 0.700 | 1.708 |
| 1AAY_E121A | 0.179 | 0.661 | 1.499 | 1.050 | 1.574 |
| 1AAY_R124A | 0.440 | 0.602 | 2.165 | 2.550 | -5.382 |
| 1B3T_R469A | 0.723 | 0.819 | 1.806 | 1.630 | -4.928 |
| 1B3T_Y518A | 0.465 | 0.438 | 1.506 | 1.520 | -1.406 |
| 1B3T_R522A | 0.703 | 0.500 | 1.397 | 2.350 | -3.896 |
| 1BPX_Y271A | 0.139 | 0.204 | 0.594 | -0.020 | -0.178 |
| 1BPX_R283A | 0.306 | 0.146 | 0.718 | 0.550 | -1.002 |
| 1BPX_E295A | 0.331 | 0.298 | 0.570 | -1.030 | -1.130 |
| 1EWQ_E41A | 0.534 | 0.671 | 0.907 | -0.870 | 1.134 |
| 1PNR_K55A | 0.124 | 0.206 | 0.738 | 0.820 | -2.374 |
| 1RUN_D138A | 0.563 | 0.247 | 0.666 | 0.020 | -1.848 |
| 1RUN_E181A | 0.164 | 0.521 | 1.518 | -0.310 | 0.562 |
| 1TN9_R5A | 0.231 | 0.175 | 1.146 | 0.900 | -0.936 |
| 1TN9_T15A | 0.509 | 0.261 | 0.500 | 0.150 | -0.002 |
| 1TN9_S18A | 0.067 | 0.228 | 0.339 | 0.570 | 0.000 |
| 1TN9_R20A | 0.095 | 0.389 | 1.408 | 1.850 | 0.000 |
| 1TN9_K21A | 0.152 | 0.078 | 0.679 | 0.490 | 0.000 |
| 1TN9_R24A | 0.819 | 0.746 | 1.044 | 1.440 | 0.000 |
| 1TN9_L26A | 0.173 | 0.646 | 0.829 | 0.430 | 0.000 |
| 1TN9_K28A | 0.920 | 0.699 | 1.778 | 1.010 | 0.000 |
| 1TN9_F38A | 0.661 | 0.661 | 1.935 | 0.740 | 0.000 |
| 1TN9_Y40A | 0.845 | 0.806 | 1.271 | 0.720 | 0.000 |
| 1TN9_W42A | 0.172 | 0.188 | 0.663 | -0.010 | 0.000 |
| 1TN9_K54A | 0.500 | 0.645 | 0.419 | 0.560 | 0.000 |
| 1TN9_R55A | 0.559 | 0.689 | 1.457 | 0.670 | 0.000 |
| 2G1P_Y184A | 0.488 | 0.490 | 1.051 | 0.920 | 0.582 |
| 3NCI_L561A | 0.113 | 0.133 | 0.707 | 0.530 | 0.056 |
| 3NCI_Y567A | 0.091 | 0.201 | 1.088 | 0.490 | 0.760 |
| 3RNU_K627A | 0.371 | 0.177 | 0.530 | -0.280 | -1.340 |
| 3UFD_Y37A | 0.286 | 0.605 | 1.448 | 0.870 | 0.442 |
| 3UFD_R46A | 0.881 | 0.644 | 0.760 | 2.070 | -1.502 |
| 3UFD_S52A | 0.222 | 0.369 | 0.599 | 0.640 | -0.402 |
| 3WTS_V170A | 0.179 | 0.138 | 0.825 | 0.160 | -0.096 |
| 4B5F_N207A | 0.389 | 0.460 | 1.018 | 0.700 | -0.664 |
| 4B5F_R208A | 0.523 | 0.613 | 1.386 | 2.500 | -1.114 |
| 4BNC_D387A | 0.170 | 0.144 | 0.861 | 0.580 | 1.282 |
| 4BNC_R391A | 0.371 | 0.677 | 1.763 | 1.960 | -4.032 |
| 4BNC_R394A | 0.500 | 0.776 | 2.055 | 1.920 | -3.804 |
| 4BNC_Y395F | 0.196 | 0.215 | 0.719 | 0.340 | 1.058 |
| 4GZN_E182A | 0.050 | 0.066 | 1.243 | -0.100 | 0.530 |
| 4HQB_W66A | 0.748 | 0.632 | 1.201 | 1.080 | -1.618 |
| 4K4G_R517A | 0.472 | 0.640 | 1.922 | 3.100 | -0.406 |
| 4TMU_S97A | 0.633 | 0.295 | 0.669 | 0.430 | -1.684 |
| 4TMU_R125A | 0.641 | 0.429 | 0.611 | 1.950 | -2.914 |
| 4TMU_R246A | 0.579 | 0.069 | 0.314 | 2.250 | -2.454 |
| 4TMU_R275A | 0.563 | 0.322 | 0.563 | 2.020 | -2.824 |
| 4TMU_T293A | 0.293 | 0.243 | 0.511 | 0.900 | -1.310 |
| 4TMU_R315A | 0.636 | 0.494 | 1.121 | 1.500 | -2.532 |
| 4TMU_W347A | 0.133 | 0.344 | 1.473 | 1.240 | -0.970 |
| 4TMU_R446A | 0.661 | 0.258 | 0.753 | 1.360 | -2.876 |
| 4ZSF_K85A | 0.205 | 0.753 | 1.878 | 2.780 | -3.944 |
| 5DWB_Q86A | 0.512 | 0.544 | 1.564 | 0.700 | 1.708 |
| 5DWB_D142A | 0.115 | 0.661 | 1.499 | 1.050 | 1.574 |
| 5DWB_D177A | 0.283 | 0.602 | 2.165 | 2.550 | -5.382 |
| 5DWB_D178A | 0.238 | 0.819 | 1.806 | 1.630 | -4.928 |
| 5DWB_D223A | 0.254 | 0.438 | 1.506 | 1.520 | -1.406 |
| 5FD3_Y536A | 0.663 | 0.500 | 1.397 | 2.350 | -3.896 |
| 5FD3_Y610A | 0.220 | 0.204 | 0.594 | -0.020 | -0.178 |
| 5TWP_H329A | 0.476 | 0.146 | 0.718 | 0.550 | -1.002 |
| 5TWP_W434A | 0.296 | 0.298 | 0.570 | -1.030 | -1.130 |
| 4XR0_H144A | 0.372 | 0.671 | 0.907 | -0.870 | 1.134 |
| 5AWH_K506A | 0.198 | 0.206 | 0.738 | 0.820 | -2.374 |

Note: The prediction results of SAMPDI, PremPDI and mCSM-NA were ∆∆G values, where the residue with ∆∆G$\geq$1.0 kcal/mol is defined as hot spots and that with ∆∆G$<$1.0 kcal/mol is defined as non-hot spot. Please note that the prediction values from these web servers have some fluctuation. The prediction results of inpPDH and PrPDH were prediction probability scores, where 0.5 was used the threshold to differentiate hot spots and non-hot spots.
